# Constrained ESKF for UAV Positioning in Indoor Corridor Environment Based on IMU and WiFi

**DOI:** 10.3390/s22010391

**Published:** 2022-01-05

**Authors:** Zhonghan Li, Yongbo Zhang

**Affiliations:** 1 School of Aeronautic Science and Engineering, Beihang University, Beijing 100191, China; Jason_lzh@buaa.edu.cn; 2 Aircraft and Propulsion Laboratory, Ningbo Institute of Technology, Beihang University, Ningbo 315100, China

**Keywords:** UAV, ESKF, WiFi, data fusion, indoor positioning

## Abstract

The indoor autonomous navigation of unmanned aerial vehicles (UAVs) is the current research hotspot. Unlike the outdoor broad environment, the indoor environment is unknown and complicated. Global Navigation Satellite System (GNSS) signals are easily blocked and reflected because of complex indoor spatial features, which make it impossible to achieve positioning and navigation indoors relying on GNSS. This article proposes a set of indoor corridor environment positioning methods based on the integration of WiFi and IMU. The zone partition-based Weighted K Nearest Neighbors (WKNN) algorithm is used to achieve higher WiFi-based positioning accuracy. On the basis of the Error-State Kalman Filter (ESKF) algorithm, WiFi-based and IMU-based methods are fused together and realize higher positioning accuracy. The probability-based optimization method is used for further accuracy improvement. After data fusion, the positioning accuracy increased by 51.09% compared to the IMU-based algorithm and by 66.16% compared to the WiFi-based algorithm. After optimization, the positioning accuracy increased by 20.9% compared to the ESKF-based data fusion algorithm. All of the above results prove that methods based on WiFi and IMU (low-cost sensors) are very capable of obtaining high indoor positioning accuracy.

## 1. Introduction

An unmanned aerial vehicle (UAV) is a drone that integrates various sensors, flight control systems, data processing systems, power systems, and other modules, which can autonomously complete specified tasks without human intervention. Since the application of UAVs from their initial military purpose, such as Predator, Global Hawk, and Black Hornet, to later civilian and commercialization purposes, such as DJI and Zero-Zero technology, the drone market has entered a fierce development state. Rotor-wing UAVs, especially quadcopters, have good development prospects in indoor environments, due to their compact structure, easy hovering, and convenient side-flight. However, GNSS signals cannot be received indoors. Because of topological structures and spatial features, the indoor environment is complex, and signals are easily blocked and reflected. Therefore, quadcopters cannot rely on GNSS to realize positioning and navigation indoors. However, pseudolite-based methods can also solve GNSS-based method problems in an indoor environment [1,2]. Considering that current indoor environments can easily receive external source signals, such as WiFi and Bluetooth, deploying pseudolite transmitters also costs a lot, and method-based pseudolites may face near–far problems and time synchronization problems [1], this article mainly discusses other indoor positioning methods.

At present, the navigation and positioning methods used in indoor quadcopters mainly include the following two categories: vision or LiDAR-based methods and external source-based methods, in which the external source includes Bluetooth, WiFi, and UWB. Among them, vision or LiDAR-based methods are the current research hotspot [3,4,5,6,7,8,9,10], and the sensors used in vision-based methods can be categorized into visual odometer [3,4], stereo camera [3,4], monocular camera [5,6,7], binocular camera [8], and optical flow sensor [9,10]. Reference [3] is mainly based on visual odometers and integrates a stereo camera and LiDAR to build an aircraft for indoor search and rescue. Based on the stereo camera and visual odometer, reference [4] additionally uses landmarks to achieve autonomous indoor navigation. A downward monocular camera and LiDAR are used to estimate the speed and attitude of a quadcopter in reference [5]. Reference [7] is based on the monocular camera and mainly improves the Oriented FAST and Rotated BRIEF (ORB) algorithm and obtains higher accuracy. The ArUco marks are used in [6] to realize indoor positioning control. The binocular camera is used to combine with IMU in [8], which realizes position and orientation control with respect to the specific target. Combining inertial sensors, optical flow, and airspeed data, reference [9] achieves indoor navigation and attitude control, while [10] realizing the tracking of a specific red line based on an optical flow sensor.

Although vision-based and LiDAR-based technology can provide high-precision indoor positioning results, in practical applications, high-precision LiDAR is expensive and obtains large amount of data, which require high calculation capabilities for drone equipment. Visual sensors are sensitive to light and dark, and processing image data also requires a large amount of calculation. Recently, with the development of mobile smart terminals, WiFi signals have become cheaper and easier to acquire. The positioning methods based on WiFi signals are less difficult to employ, and less is spent on construction. In addition, WiFi-based positioning methods are widely used in the indoor pedestrian positioning field. The common WiFi positioning approaches include fingerprinting [11,12,13,14,15,16,17,18], trilateration [19], and their combination [20]. Fingerprinting is more widely used, because location information can be provided without knowing access point locations and propagation models. Reference [11] proposes a Weighted K-Nearest Neighbor (WKNN) algorithm based on spatial feature division, which improves the smoothness of estimation results. Reference [12] uses the Local Feature-based Deep Long Short-Term Memory approach (LF-DLSTM) and achieved in local positioning. References [13,14] are specific applications for indoor intelligent vehicles and pedestrians, respectively; the errors of both are meter level. Series of studies on fingerprint optimization are in [15,16,17,18]. Reference [17] focuses on increasing the interference robustness of WiFi fingerprints, and the least square method is used to reduce location error in [18]. Reference [21] optimizes the AdaBoost algorithm to increase the accuracy of indoor/outdoor detection. The WiFi signature map and continuous perceptual model are established in [22] to realize indoor mobile robot navigation, and the localization error is a mean of 1.2 m. The series of references indicate that the WiFi-based positioning algorithm can meet the requirements of indoor positioning for mobile robots. In this article, a set of WiFi positioning methods is proposed which fuses IMU and WiFi by the constrained Error-State Kalman Filter (ESKF) method, and the accuracy of positioning becomes greatly improved.

The rest of this article is organized as follows. In Section 2, the WiFi-based and IMU-based positioning methods are presented. In Section 3, the ESKF-based data fusion method and probability-based optimization method are presented. In Section 4, we provide experimental results of WiFi-based positioning, IMU-based positioning, ESKF-based data fusion method, and constrained optimization results. Finally, in Section 5, we draw conclusions.

## 2. Positioning Methods Based on IMU and WiFi

### 2.1. WiFi-Based Positioning Method

**Figure 1:**
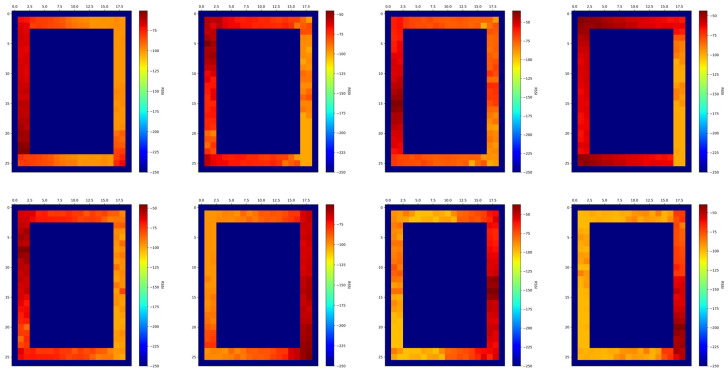
WiFi signal strength heatmap of 8 different APs.

WiFi-based indoor positioning algorithms, especially the WKNN algorithm, have some inherent problems. On the one hand, before realizing positioning, data preprocessing is required, which needs to collect WiFi fingerprint data, and the number and the Media Access Control (MAC) address of reference points (RPs) are different. However, in order to facilitate the matching of fingerprint vectors and the calculation of the Euclidean distance, we need to extract the same access point (AP) information shared by all RPs into one database. During this process, part of the original data is lost. When the target area is small, the loss of data does not have a significant impact on the location accuracy. However, when the target area is large or the spatial occlusion becomes serious, the APs in the database are not enough to support location, and there are even no fingerprint data in some places. On the other hand, the WKNN algorithm ignores the influence of the k points closest to the current position. When in the location process, it matches from the global area and only considers the constraints brought by the Euclidean distance. In addition, in the actual corridor environment, the signal strength in same position will be affected by different APs on the same or different floors. There are eight different AP signal strength heatmaps of one corridor area in Figure 1. The data were received in the true corridor environment. From the heatmaps we find that it is difficult to establish the WiFi propagation model to realize positioning. As a result, the algorithm we used is based on spatial feature partition and the WKNN algorithm and improved in order to achieve better location results.

#### 2.1.1. Zone Partition Algorithm by Spatial Feature

**Figure 2:**
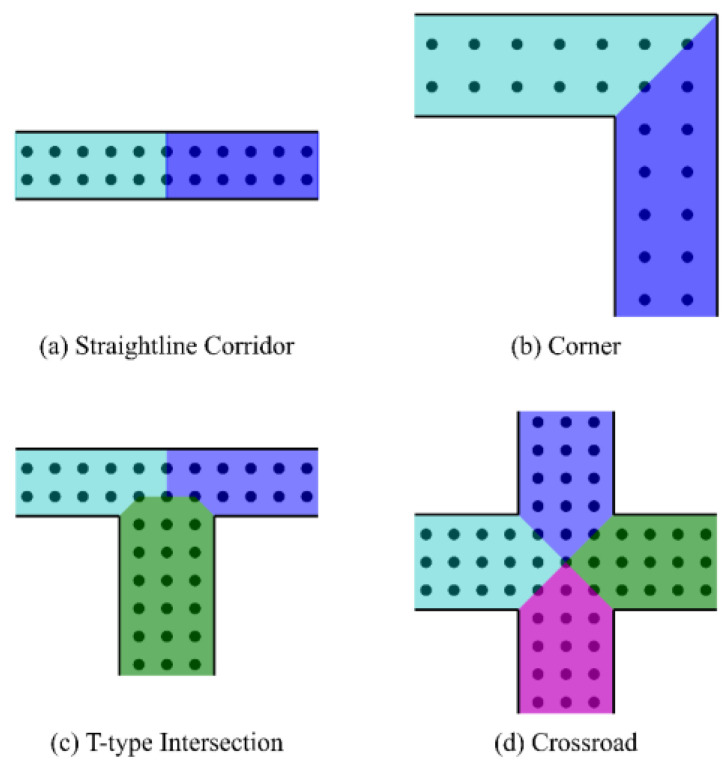
Some partition methods.

Research was conducted on the WKNN algorithm based on zone partition [11], and this method references the research. The WiFi positioning method based on spatial features can reduce the loss of original data and obtain higher accuracy. In the partitioning process, it is necessary to ensure that two adjacent areas share the same boundary points to achieve seamless partitioning. In the indoor environment, there are mainly four types in the corridor environment, which are straight line, corner, T-type, and crossroad as shown in Figure 2.

After establishing the WiFi fingerprint database for each partition, it is particularly important to accurately identify to which zone the UAV belongs. The zone discrimination algorithm used in this article contains two criteria, namely, Diagnostic Sequence Matching and the Euclidean Distance Comparison. Firstly, the diagnostic sequence needs to be generated, which is compared with the real-time sequence obtained by UAV, in order to obtain a preliminary discrimination result. The algorithm to obtain the diagnostic sequence is as follows.

For the *i*-th zone, sum the received signal strength indication (RSSI) of the same MAC address in the database and sort results from large to small. The result is as follows:

(1) $${\left[{}_{i}sum^{1}{,}_{i}sum^{2}{,}_{i}sum^{3},\cdots_{i}sum^{n_{i}}\right]}^{T}$$

where

(2) $${}_{i}sum^{j}=\sum_{k=1}^{m}{}_{i}RSSI_{j}^{k},(j=1,2,\cdots n_{i})$$

$n_{i}$ is the number of the MAC address in the database of the *i*-th zone. ${}_{i}sum^{j}$ means the sum of the RSSI of the *j*-th MAC address in the *i*-th zone, and *m* is the number of received RSSI of the *j*-th MAC address in the *i*-th zone.

Extract the former q elements of Formula (1), and their corresponding MAC address constitute the diagnostic sequence

(3) $${\left[{}_{i}MAC^{1}{,}_{i}MAC^{2}{,}_{i}MAC^{3},\cdots {,}_{i}MAC^{q}\right]}^{T}$$

However, at the junction of two adjacent zones, only relying on Diagnostic Sequence Matching may not be able to accurately determine the current zone of UAV. Thus, we need the Euclidean Distance Comparison to obtain precise results. The real-time RSSI vector obtained by UAV in real flight is

(4) $${\left[rssi_{1},rssi_{2},rssi_{3},\cdots ,rssi_{p}\right]}^{T}$$

and the vectors in *i*-th zone and *j*-th zone are

(5) $${\left[{}_{i}RSSI_{k_{i}}^{1}{,}_{i}RSSI_{k_{i}}^{2},\cdots {,}_{i}RSSI_{k_{i}}^{n_{k_{i}}}\right]}^{T},{\left[{}_{j}RSSI_{k_{j}}^{1}{,}_{j}RSSI_{k_{j}}^{2},\cdots {,}_{j}RSSI_{k_{j}}^{n_{k_{j}}}\right]}^{T}$$

The Euclidean distance between Formulae (4) and (5) should be calculated one by one, and the distance set can be obtained as

(6) $$\left\{\begin{array}{cc} L_{i}^{1} & L_{j}^{1}\\ L_{i}^{2} & L_{j}^{2}\\ \vdots & \vdots \\ L_{i}^{m_{i}} & L_{j}^{m_{j}} \end{array}\right\}$$

Then calculate the average value of the Euclidean distance, and the zone with the smaller value is the target.

#### 2.1.2. WKNN Algorithm Based on Zone Partition

In the zone, the WKNN algorithm is used to realize UAV positioning. The algorithm contains two stages: the offline stage and online stage. In the offline stage, the WiFi fingerprint database is established; while in the online stage, the UAV acquires the RSSI of the current position and calculates the Euclidean distance of the real-time RSSI and database one. The position with the minimum distance is the target.

The data format in the database is

(7) $$\left(\begin{array}{cc} x_{1} & y_{1}\\ x_{2} & y_{2}\\ \vdots & \vdots \\ x_{n} & y_{n} \end{array}|\begin{array}{cccc} RSSI_{1}^{1} & RSSI_{1}^{2} & \cdots & RSSI_{1}^{m}\\ RSSI_{2}^{1} & RSSI_{2}^{2} & \cdots & RSSI_{2}^{m}\\ \vdots & \vdots & \ddots & \vdots \\ RSSI_{n}^{1} & RSSI_{n}^{2} & \cdots & RSSI_{n}^{m} \end{array}\right)$$

where $\left(x_{i},y_{i}\right)$ is the position coordinates of the *i*-th RP. $RSSI_{i}^{j}$ is the received WiFi signal strength indication of the *j*-th AP of the *i*-th RP. *n* is the number of RPs, and *m* is the number of APs.

During the movement, the RSSI of the current position, which is obtained in real time, is

(8) $$T=(rssi_{1},rssi_{2},rssi_{3},\cdots rssi_{m})$$

The Euclidean distance is used to characterize the distance between two vectors, which are the real-time measured RSSI and that in the database, that is

(9) $$d_{i}=\left\|T_{i}-R_{i}{}^{j}\right\|=\sqrt{\sum_{j=1}^{m}{\left|T_{j}-R_{i}{}^{j}\right|}^{2}}$$

where $d_{i}$ is the distance between two vectors. $T_{j}$ is the signal strength vector of the *j*-th AP and is measured in realtime, and $R_{i}^{j}$ is the signal strength vector of the *j*-th AP in *i*-th RP in database.

Then utilize the WKNN algorithm, rank $d_{i}$ from small to large, select the smallest *k* points as the reference points for position estimation, and record it as the reference point set D, as shown in Formula (10).

(10) $$D=\{\begin{array}{cc} \left(x_{i},y_{i},R_{i}\right)| & i=1,2,3\cdots K \end{array}\}$$

The reciprocal of the Euclidean distance $d_{i}$ determines the weight, and the current position $\left(\hat{x},\hat{y}\right)$ in zone is located by Formula (11).

(11) $$\left(\hat{x},\hat{y}\right)=\frac{\sum_{i=1}^{K}\frac{\left(x_{i},y_{i}\right)}{D_{i}}}{\sum_{i=1}^{K}\frac{1}{D_{i}}}$$

### 2.2. IMU-Based Positioning Algorithm

The inertial navigation system (INS), which is based on IMU sensors, measures the acceleration of vehicles and performs integration operations to obtain the current speed and position of the UAV. The INS equipment is installed in the vehicle, does not rely on external information or radiate energy to the outside world and is not easily interfered. However, due to the double integration operations, the system has obvious accumulated errors.

The basis of inertial navigation is a high-precision measuring element, generally the accelerometer and gyroscope, and the accelerometer measurement model can be expressed as

(12) $$a_{m}^{b}=a^{b}+b_{a}+n_{a}$$

where $a^{b}\in {\mathbb{R}}^{3}$ is the true value of the specific force in the body coordinate system. $b_{a}$ is the drift error, and $n_{a}$ is the accelerometer noise vector, which is regarded as Gaussian white noise. Further, the drift error $b_{a}$ can be modeled as

(13) $${\dot{b}}_{a}=n_{b_{a}}$$

where $n_{b_{a}}$ is regarded as Gaussian white noise.

The gyroscope is a sensor based on Coriolis force, and its measurement model can be expressed as

(14) $$w_{m}^{b}=w^{b}+b_{g}+n_{g}$$

where $w^{b}\in {\mathbb{R}}^{3}$ is the true value of angular velocity in the body coordinate system. $b_{g}$ is the drift error, and $n_{g}$ is the gyroscope noise vector, also regarded as Gaussian white noise. Further, the drift error can be modeled as

(15) $${\dot{b}}_{g}=n_{b_{g}}$$

where $n_{b_{g}}$ is regarded as Gaussian white noise.

The drone position vector is composed of plane coordinates and height. In this article, we controlled the drone height as a constant, focusing on the 2D position, so the UAV position vector $p^{e}$ is simplified as

(16) $$p^{e}≜{\left[p_{x_{e}},p_{y_{e}}\right]}^{T}\in {\mathbb{R}}^{3}$$

combined with the accelerometer measurement model, considering the integral relationship between position and velocity, the INS estimation formula can be expressed as

(17) $$\{\begin{array}{c} {\dot{p}}^{e}=v^{e}\\ {\dot{v}}^{e}=R(a_{m}^{b}-b_{a}-n_{a})+ge_{3}\\ {\dot{b}}_{a}=n_{b_{a}} \end{array}$$

where $b_{a}$ is accelerometer drift error, and $R≜R_{b}^{e}$ is the rotation matrix characterizing body coordinate to global coordinate, which can be calculated as

(18) $$R_{b}^{e}=\left[\begin{array}{ccc} c\theta c\psi & c\psi s\theta \sin\varphi -s\psi c\varphi & c\psi s\theta c\varphi +s\psi s\varphi \\ c\theta s\psi & s\psi s\theta \sin\varphi +c\psi c\varphi & s\psi s\theta c\varphi -c\psi s\theta \\ -s\theta & s\varphi c\theta & c\varphi c\theta \end{array}\right]$$

where *c* is cos, *s* is sin. θ is pitch angle. φ is roll angle, and ψ is yaw angle.

## 3. Data Fusion Method and Optimization

This section may be divided by subheadings. It should provide a concise and precise description of the experimental results, their interpretation, as well as the experimental conclusions that can be drawn.

### 3.1. ESKF Algorithm for Combined Positioning

The ESKF method, which used an error-state representation, has several benefits compared to a nominal state representation [21]. First of all, the orientation error-state is represented as a 3D vector, which makes the state parameters equal to the degrees of freedom. Secondly, the value of error-state parameters is always around zero, which makes the computation of the Jacobians easy and fast. Finally, singularities, gimbal clock issues, or similar problems can be avoided because of the small value of the orientation error-state.

#### 3.1.1. System States

In the ESKF method, there are three states to describe the system, which are true-state $x_{t}$, nominal state x, and error-state δx and satisfy

(19) $$x_{t}=x⊕\delta x$$

here ⊕ indicates a generic composition. In all three states, the state vector contains

- p: position of UAV in global frame;
- v: velocity of UAV in global frame;
- q: quaternion of UAV;
- R: rotation matrix of UAV;
- $a_{b}$: accelerometer bias;
- $w_{b}$: gyroscope bias.

For instance, the variable p in the true, nominal, and error-states should be expressed as $p_{t},p,\delta p$. In addition, note that in the error-state, the angular error δθ is used to describe the rotation error-state, such as quaternions or rotation matrix is used to describe rotation. The relationships between them are $\delta q=e^{\delta \theta /2},\delta R=e^{{\left[\delta \theta \right]}_{\times }}$.

#### 3.1.2. System Kinematics Models

The system true-state kinematics model is as follows.

(20) $${\dot{p}}_{t}=v_{t}$$

(21) $${\dot{v}}_{t}=a_{t}$$

(22) $${\dot{q}}_{t}=\frac{1}{2}q_{t}⊗w_{t}$$

(23) $${\dot{a}}_{bt}=a_{w}$$

(24) $${\dot{w}}_{bt}=w_{w}$$

where the noisy accelerometer’s reading in the body frame makes up the true acceleration $a_{t}$. Similarly, the true angular rate $w_{t}$ is obtained from a gyroscope with noise in the body frame. So they satisfy

(25) $$a_{t}=R_{t}(a_{m}-a_{bt}-a_{n})+g_{t}$$

(26) $$w_{t}=w_{m}-w_{bt}-w_{n}$$

where $a_{n},w_{n}$ denote the accelerometer and gyroscope noise, and $a_{w},w_{w}$ are the Gaussian random walk noise of biases.

Similar to the true-state kinematics model, the nominal-state kinematics model is without noises or perturbations:

(27) $$\dot{p}=v$$

(28) $$\dot{v}=R(a_{m}-a_{b})+g$$

(29) $$\dot{q}=\frac{1}{2}q⊗(w_{m}-w_{b})$$

(30) $${\dot{a}}_{b}=0$$

(31) $${\dot{w}}_{b}=0$$

The error-state kinematics model is derived from the nominal-state kinematics model,

(32) $$\delta \dot{p}=\delta v$$

(33) $$\delta \dot{v}=-R{\left[a_{m}-a_{b}\right]}_{\times }\delta \theta -R\delta a_{b}+\delta g-Ra_{n}$$

(34) $$\delta \dot{\theta }=-{\left[w_{m}-w_{b}\right]}_{\times }\delta \theta -\delta w_{b}-w_{n}$$

(35) $$\delta {\dot{a}}_{b}=a_{w}$$

(36) $$\delta {\dot{w}}_{b}=w_{w}$$

where the accelerometer noise is assumed to be white, uncorrelated, and isotropic. However, the assumption cannot be made in cases where XYZ accelerometers are not identical.

#### 3.1.3. Propagation

The propagation step contains estimate error-state propagation and error covariance propagation. The estimate error-state is propagated through the error-state kinematics model in discrete time, which is

(37) $$\delta p_{k+1}=\delta p_{k}+\delta v_{k}\Delta t$$

(38) $$\delta v_{k+1}=\delta v_{k}+(-R_{k}{\left[a_{m,k}-a_{b,k}\right]}_{\times }\delta \theta_{k}-R_{k}\delta a_{b,k}+\delta g)\Delta t+v_{i}$$

(39) $$\delta \theta_{k+1}=R_{k}^{T}\{(w_{m,k}-w_{b,k})\Delta t\}\delta \theta_{k}-\delta w_{b,k}\Delta t+\theta_{i}$$

(40) $$\delta a_{b,k+1}=\delta a_{b,k}+a_{i}$$

(41) $$\delta w_{b,k+1}=\delta w_{b,k}+w_{i}$$

where

(42) $$v_{i}=\sigma_{a_{n}}^{2}\Delta t^{2}I$$

(43) $$\theta_{i}=\sigma_{w_{n}}^{2}\Delta t^{2}I$$

(44) $$a_{i}=\sigma_{a_{w}}^{2}\Delta tI$$

(45) $$w_{i}=\sigma_{w_{w}}^{2}\Delta tI$$

Now the error-state system can be summarized as

(46) $$\delta {\hat{x}}_{k+1}=f(x,\delta x,u_{m},i)=F_{x}(x,u_{m})\delta \hat{x}$$

(47) $$F_{x}=\left[\begin{array}{cccccc} I & I\Delta t & 0 & 0 & 0 & 0\\ 0 & I & -R_{k}{\left[a_{m,k}-a_{b,k}\right]}_{\times }\Delta t & -R\Delta t & 0 & I\Delta t\\ 0 & 0 & R_{k}^{T}\{(w_{m,k}-w_{b,k})\Delta t\} & 0 & -I\Delta t & 0\\ 0 & 0 & 0 & I & 0 & 0\\ 0 & 0 & 0 & 0 & I & 0 \end{array}\right]$$

The error covariance propagation is

(48) $${\hat{P}}_{k+1}=F_{x,k}P_{k}F_{x,k}^{T}+F_{i}Q_{i}F_{i}^{T}$$

(49) $$F_{i}=\left[\begin{array}{cccc} 0 & 0 & 0 & 0\\ I & 0 & 0 & 0\\ 0 & I & 0 & 0\\ 0 & 0 & I & 0\\ 0 & 0 & 0 & I\\ 0 & 0 & 0 & 0 \end{array}\right],\;Q_{i}=\left[\begin{array}{cccc} v_{i} & 0 & 0 & 0\\ 0 & \theta_{i} & 0 & 0\\ 0 & 0 & a_{i} & 0\\ 0 & 0 & 0 & w_{i} \end{array}\right]$$

$F_{x}$ and $F_{i}$ are the Jacobians of f() with respect to the error and perturbation vectors, and $Q_{i}$ is the covariance matrix of the perturbation impulses.

#### 3.1.4. Measurement Update

A WiFi position error measurement δy is used to update the error-state vector δx.

(1) Observation Model: with error-state representation, the observation model is simply linear:

(50) $$\delta y=H\delta x=\left[\begin{array}{cc} I_{2} & 0\\ 0 & 0 \end{array}\right]\delta x$$

(2) Correction: after the WiFi measurements are computed, the filter corrections are implemented the same as the KF filter.

(51) $$K={\hat{P}}_{k+1}H_{k}^{T}{\left(H{\hat{P}}_{k+1}H^{T}+V\right)}^{-1}$$

(52) $$\delta x_{k+1}=K(\delta y_{k+1}-H\delta {\hat{x}}_{k+1})$$

(53) $$P_{k+1}=(I-KH){\hat{P}}_{k+1}$$

where V is the covariance of the measurements’ white Gaussian noise, and the Jacobian matrix H is defined as

(54) $$H={\frac{\partial h}{\partial \delta x}|}_{x}$$

#### 3.1.5. Nominal State Update

After the error-state update, the nominal-state is updated with the observed error-state using the appropriate compositions,

(55) $$x_{k+1}=x_{k}⊕\delta {\hat{x}}_{k}$$

that is,

(56) $$p_{k+1}=p_{k}+\delta {\hat{p}}_{k}$$

(57) $$v_{k+1}=v_{k}+\delta {\hat{v}}_{k}$$

(58) $$q_{k+1}=q_{k}⊗q\{\delta {\hat{\theta }}_{k}\}$$

(59) $$a_{b,k+1}=a_{b,k}+\delta {\hat{a}}_{b,k}$$

(60) $$w_{b,k+1}=w_{b,k}+\delta {\hat{w}}_{b,k}$$

#### 3.1.6. ESKF Reset

After error integrating into the nominal-state, the error-state variables need to be reset. This can be written as follows:

(61) $$\delta x_{k+1}=\delta x_{k}⊖\delta {\hat{x}}_{k}$$

where ⊖ stands for the compositive inverse of ⊕, and the estimate error-state variables are set to 0. Finally, to make the ESKF update complete, the covariance of the error needs to be updated as follows:

(62) $$P_{k+1}=G_{k}P_{k}G_{k}^{T}$$

### 3.2. Optimization by Constrained ESKF

Computed by the ESKF algorithm, the IMU-based state and the WiFi-based state are fused together. However, the fusion data are not accurate enough for UAV indoor navigation. Due to specific features of the corridor environment, there are position constraints that can be used to improve positioning accuracy. The constraints can be divided into equality constraints and inequality constraints in the filtering field. For equality constraints, we can simplify the model variables utilizing the equations or implement state extensions to settle filtering problems. For inequality constraints, there are several methods mainly based on projection and probability. By projecting the filter results onto the constraint surface, the projection-based method can solve the inequality constraints problem. By assuming the filter results obey Gaussian distribution and cutting the distribution function at the boundary of constraints, the problem can also be solved.

#### 3.2.1. Position Constraints

**Figure 3:**
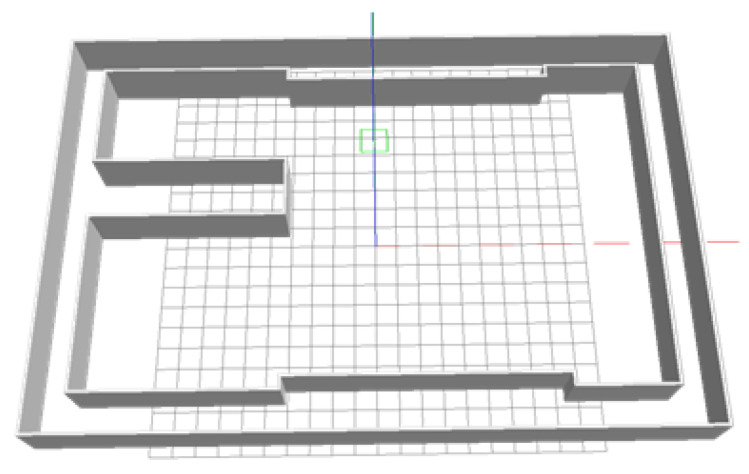
Gazebo simulation model of indoor corridor environment.

Unlike the cluttered indoor environment, the spatial feature of a corridor is simple, and specific physical constraints exist because of the corridor walls. The indoor corridor environment is shown in Figure 3 in the form of gazebo simulation model.

**Figure 4:**
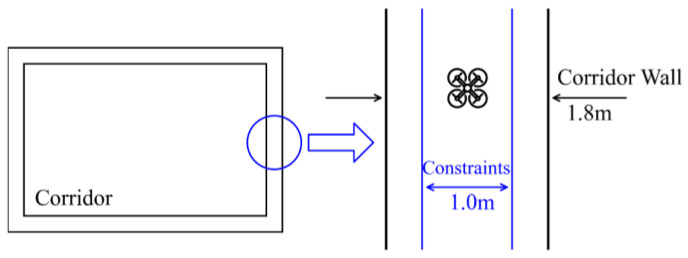
Simplified diagram of indoor corridor environment and its constraints.

Only considering 2D position constraints, the corridor environment can be simplified as Figure 4, and the constraints are shown in the right of the figure. We implemented a 1 m constraint based on the center line in the 1.8 m corridor, and the constraints can be expressed by (46).

(63) a≤x≤b

where vector x represents the UAV position in the corridor environment, and variables a,b represent the lower and upper bounds of the position constraints.

#### 3.2.2. Optimization Algorithm Based on Probability

With the physical constraints in indoor corridor environment, we assumed that at time interval k we can obtain s state constraints,

(64) $$a_{ki}\leq \Phi_{ki}^{T}x_{k}\leq b_{ki}\;i=1,2,\cdots ,s$$

and this is a bilateral constraint of the state linear function $\Phi_{ki}^{T}x_{k}$. Then, based on the filter’s state estimation ${\hat{x}}_{k}$ and its covariance $P_{k}$, we performed a transformation as follows:

(65) $$z_{ki}=\rho W^{-1/2}T^{T}(x_{k}-{\tilde{x}}_{ki})$$

where ρ is a n×n orthogonal matrix obtained by Gram–Schmidt Orthogonalization. Matrices T,W are derived from the Jordanian Specification Decomposition of ${\tilde{P}}_{ki}$, and ${\tilde{x}}_{ki}$ represents the state estimation that satisfies the first *i* constraints, and ${\tilde{P}}_{ki}$ is the covariance of ${\tilde{x}}_{ki}$.

So the upper bound can transform into

(66) $$\left[\begin{array}{cccc} 1 & 0 & \cdots & 0 \end{array}\right]z_{ki}\leq \frac{b_{ki}-\Phi_{ki}^{T}{\tilde{x}}_{ki}}{{\left(\Phi_{ki}^{T}{\tilde{P}}_{ki}\Phi_{ki}\right)}^{1/2}}\leq d_{ki}$$

similarly, the lower bound can transform into

(67) $$\left[\begin{array}{cccc} 1 & 0 & \cdots & 0 \end{array}\right]z_{ki}\geq \frac{a_{ki}-\Phi_{ki}^{T}{\tilde{x}}_{ki}}{{\left(\Phi_{ki}^{T}{\tilde{P}}_{ki}\Phi_{ki}\right)}^{1/2}}\geq c_{ki}$$

then we obtain a normalized scalar constraint,

(68) $$c_{ki}\leq \left[\begin{array}{cccc} 1 & 0 & \cdots & 0 \end{array}\right]z_{ki}\leq d_{ki}$$

Remove the part beyond the constraint in the Gaussian probability function and calculate the rest probability function and transform the cut-off probability into region area of 1, then we obtain

(69) $$pdf(\xi )=\{\begin{array}{l} \alpha \exp(-\xi^{2}/2),\;\xi \in [c_{ki},d_{ki}]\\ 0,\;\;\;\;\;others \end{array}$$

where

(70) $$\alpha =\frac{\sqrt{2}}{\sqrt{\pi }[erf(d_{ki}/2)-erf(c_{ki}/2)]}$$

and erf(·) is the error function

(71) $$erf(t)=\frac{2}{\sqrt{\pi }}\int_{0}^{t}\exp(-\gamma^{2})dr$$

After adding the constraint, the mean and variance of the state estimate are

(72) $${\tilde{z}}_{k,i+1}={\left[\begin{array}{cccc} \mu & 0 & \cdots & 0 \end{array}\right]}^{T}$$

(73) $$Cov({\tilde{z}}_{k,i+1})=(\sigma^{2},1,\cdots ,1)$$

where

(74) $$\mu =\alpha [\exp(-c_{ki}^{2}/2)-\exp(-d_{ki}^{2}/2)]$$

(75) $$\sigma^{2}=\alpha [\exp(-c_{ki}^{2}/2)(c_{ki}-2\mu )-\exp(-d_{ki}^{2}/2)(d_{ki}-2\mu )]+\mu^{2}+1$$

**Figure 5:**
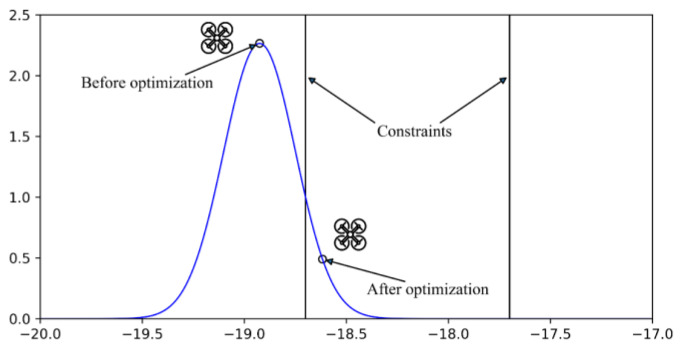
Schematic diagram of constrained algorithm based on probability.

Then perform an inverse transformation. We can obtain the state estimation and covariance that meets the first constraint. Repeat i−1 times, and we obtain a state estimation. Figure 5 shows the schematic diagram of constrained algorithm by one real positioning result in experiments.

(76) $${\tilde{x}}_{k,i+1}=TW^{1/2}\rho^{T}{\tilde{z}}_{k,i+1}+{\tilde{x}}_{ki}$$

(77) $${\tilde{P}}_{k,i+1}=TW^{1/2}\rho^{T}Cov({\tilde{z}}_{k,i+1})\rho W^{1/2}T^{T}$$

## 4. Experiment Results and Discussion

The indoor aircraft platform is independently designed to conduct a series of experiments, which are the WiFi-based and IMU-based positioning methods, the ESKF-based combined positioning method, and the constrained ESKF method for optimization. In this chapter, after introducing the platform and testing environment, we show the detailed results of all the above experiments.

### 4.1. UAV Platform and Testing Environment

**Figure 6:**
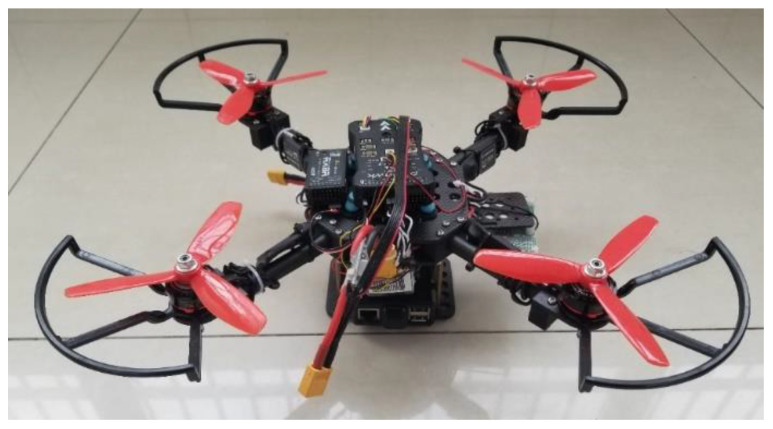
Indoor aircraft platform.

Figure 6 is the quadcopter we designed for the indoor corridor environment. It includes a Pixhawk flight controller, an onboard computer Raspberry Pi 3B+, and an optical flow sensor. The stability of the basic flight of the quadcopter is guaranteed by a flight controller. DroneKit project was used to control the copter’s movement. In addition, note that the optical flow only participated in maintaining fixed-point flight, not in positioning and navigation. Before real flight, we carried out a series of experiments to guarantee feasibility. The simulation model we used is SITL, whose biggest advantage is that the simulated sensor data can be obtained directly from the existing flight dynamics model library. And the simulation experiment platform includes SITL, MAVProxy, and Mission Planner.

**Figure 7:**
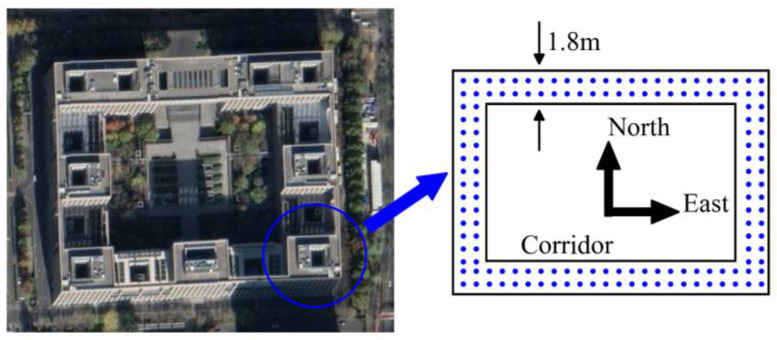
Experiment sites and its simplified diagram.

The simulated experiment site is the same as the site where the fingerprint database was established before as shown in Figure 7. The square corridor is roughly a 20 m×30 m rectangle with a corridor width of 1.8 m.

### 4.2. WiFi-Based and IMU-Based Positioning Experimnet Results and Discussion

#### 4.2.1. WiFi-Based Positioning Experiment Results and Discussion

The square grid was used as a reference to build the fingerprint database as shown in Figure 7. The reference points are separated by 1.2 m, and 158 reference points were arranged in the corridor. Considering that different receiving directions may cause interference to the WiFi signal strength, we took 10 measurements in each of the four directions at each reference point and then took the average of the measurement values as the signal strength and stored it in the fingerprint database.

**Figure 8:**
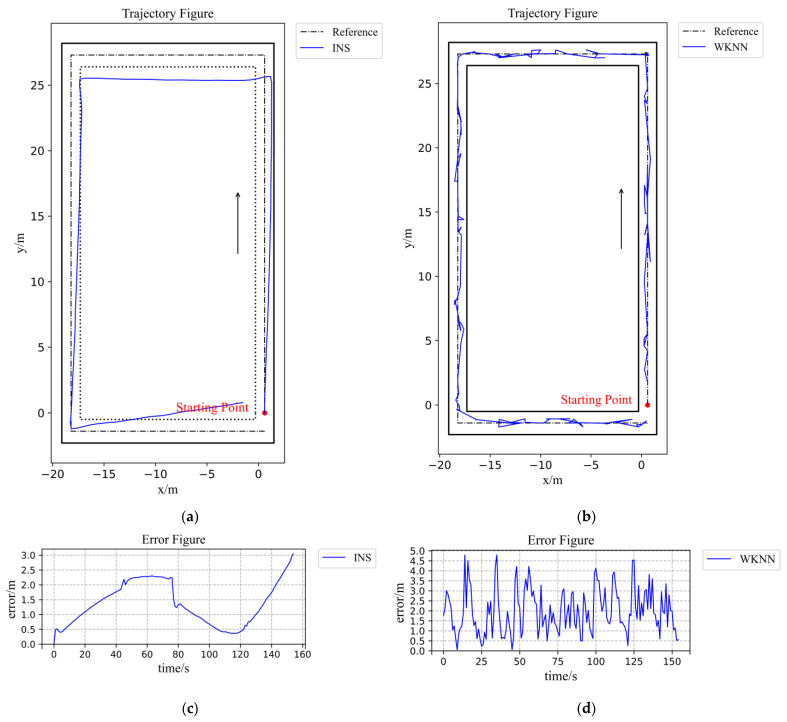
Figures of IMU-based and WiFi-based experiments results: (**a**) Figure of IMU-based positioning trajectory; (**b**) Figure of WiFi-based positioning trajectory. (**c**) Error figure of IMU-based positioning trajectory; (**d**) Error figure of WiFi-based positioning trajectory.

The WiFi positioning experiment results are shown in Figure 8 and Table 1. In the trajectory figure, the black line is the corridor wall, the dotted line is the reference trajectory, and the blue line is zone partition-based WKNN trajectory. The positioning trajectory does not hit the wall, and the smoothness is very poor. The data points are moving together and jumping back and forth, which means in the further navigation part that the stability of the actual UAV flight cannot be satisfied. With a maximum error of 4.79 m, removing a minimum error of 0.01 m, a zone partition-based WKNN algorithm can reach an average error of 1.98 m.

#### 4.2.2. IMU-Based Positioning Experiment Results and Discussion

The IMU-based positioning experiment results are shown in Figure 8 and Table 2. In Figure 8a, the dotted line is the reference trajectory, and the blue line is the IMU-based trajectory, which exhibits an obvious cumulative error due to the two integrations. Only by realizing the IMU-based positioning, the experiment result deviates from the reference trajectory too much. Although it is smoother than the WiFi-based positioning method, the UAV cannot finish the whole flight safely. With a maximum error of 3.06 m, removing a minimum error of 0.001 m, the IMU-based positioning algorithm can reach a mean error of 1.37 m.

### 4.3. ESKF for Combined Positioning Method Experiment Result and Discussion

**Figure 9:**
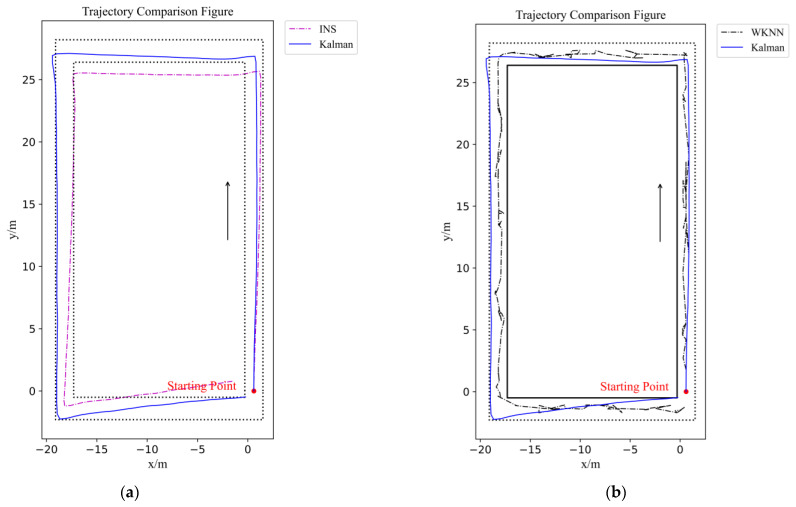

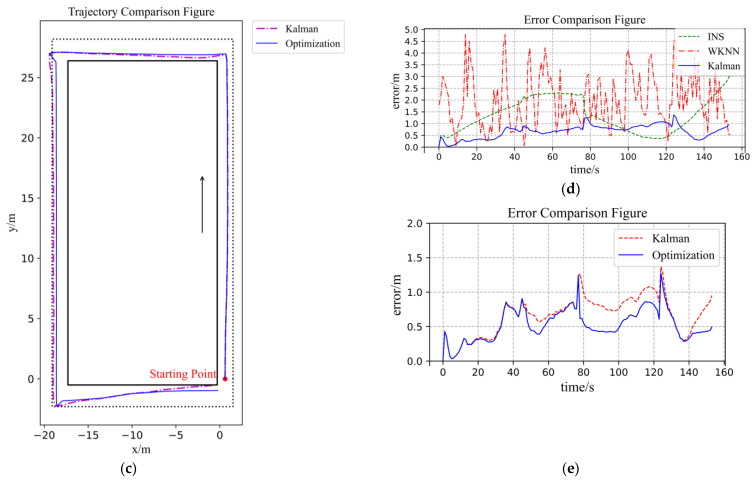
Figures of ESKF-based and constrained ESKF experiment results and comparison figures: (**a**) Figure of IMU-based and ESKF positioning trajectory comparison; (**b**) Figure of WiFi-based and ESKF positioning trajectory comparison; (**c**) Figure of ESKF and constrained ESKF positioning trajectory comparison; (**d**) Error figure of three positioning methods; (**e**) Error figure of data fusion method and optimization positioning method.

The ESKF result is shown in Figure 9 and Table 3, and the comparisons among all three algorithms are shown in Figure 9d. After the ESKF data fusion, the average value of positioning deviation decreased to 0.67 m, and the positioning accuracy improved by 51.09% compared to the IMU-based algorithm and 66.16% compared to the WiFi-based algorithm. The maximum error also decreased significantly; it decreased by 55.23% compared to the IMU-based algorithm and by 71.4% compared to the WiFi-based algorithm. The positioning accuracy is evidently enhanced through the ESKF data fusion algorithm. In terms of the positioning trajectory, the ESKF-based data fusion result combines specific features of the other two algorithms, which made the combined trajectory smoother and closer to the reference line. However, because there was an obvious drift with the IMU sensors, the combined trajectory partly overlapped with the corridor walls, which will bring risk in further indoor navigation control.

### 4.4. Constrained ESKF Positioning Experiment Result and Discussion

The constrained ESKF optimization result is shown in Figure 9. In addition, the comparison of the two algorithm errors is shown in Table 4. The optimization operation effectively increased the accuracy of the ESKF-based combined positioning method. The maximum error decreased by 6.57%, and the positioning accuracy improved by 20.9%. With a mean error of 0.53 m and a maximum error of 1.28 m, the UAV positioning accuracy obtained great protection.

**Figure 10:**
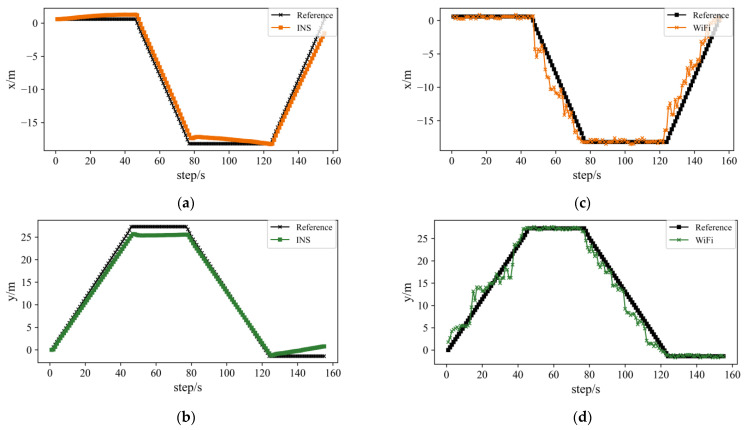

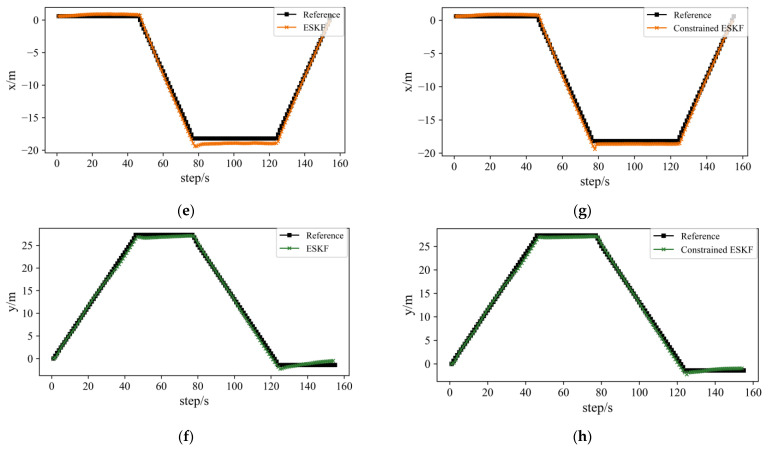
Figures of all methods comparison with reference trajectory: (**a**,**b**) are comparison figures of IMU-based method; (**c**,**d**) are comparison figures of WiFi-based method; (**e**,**f**) are comparison figures of ESKF-based data fusion method; (**g**,**h**) are comparison figures of constrained ESKF method.

Figure 10 is a summary of the set of indoor corridor environment positioning methods, which are the WiFi-based and IMU-based positioning methods, the ESKF-based data fusion method, and the probability-based constrained method. From (a), (b) and (c), and (d) we can easily see the deviation caused by IMU sensors drift and fluctuation in the WiFi-based positioning method. After the ESKF-based data fusion method, the accuracy improved significantly. After implementing the probability-based constrained method, the positioning results further improved.

## 5. Conclusions

With the progress of urbanization, the area of buildings is becoming larger and larger, there are more and more giant buildings, and the market demand for indoor drones is also increasing. Due to its light weight, small size, and flexible motion, indoor drones can easily adapt to narrow spaces, and are of great significance for completing tasks, such as indoor reconnaissance, indoor rescue, and target pickup. At present, the research hotspots of indoor drone navigation mainly focus on visual technology and SLAM technology, and there are few studies on WiFi-based drone navigation and positioning methods. Based on the indoor positioning technology of pedestrians and combining the characteristics of the indoor environment, we proposed a positioning method based on the fusion of WiFi and IMU measurements and obtained good experimental results.

Firstly, we carry out a series of simulation experiments for the IMU-based and WiFi-based positioning methods and obtained an average error of 1.98 m for WiFi-based positioning and an average error of 1.37 m for IMU-based system. There are specific features among them. The IMU-based positioning method can obtain smoother results but deviates greatly from the reference trajectory. WiFi-based positioning can obtain closer results to the reference trajectory, but the data jump back and forth, which cannot guarantee the stability of further navigation control. The ESKF-based data fusion method improved the positioning accuracy by 51.09% compared with the IMU-based method and by 66.16% compared with the WiFi-based method. To further improve accuracy, the probability-based optimization method is realized, which improves the accuracy of positioning by 20.9%.

## Figures and Tables

**Table 1:** Errors of WiFi positioning algorithm.

Algorithm	Min/m	Max/m	Mean/m
Zone partition-based WKNN algorithm	0.01	4.79	1.98

**Table 2:** Errors of INS positioning algorithm.

Algorithm	Min/m	Max/m	Mean/m
IMU-based positioning algorithm	0.001	3.06	1.37

**Table 3:** Comparisons of errors of three algorithms.

Algorithm	Min/m	Max/m	Mean/m
WiFi-based positioning algorithm	0.01	4.79	1.98
IMU-based positioning algorithm	0.001	3.06	1.37
ESKF-based data fusion algorithm	0.001	1.37	0.67

**Table 4:** Comparisons of errors of three algorithms.

Algorithm	Min/m	Max/m	Mean/m
ESKF-based data fusion algorithm	0.001	1.37	0.67
Constrained ESKF optimization algorithm	0.001	1.28	0.53

## Data Availability

Not applicable.
